# Mitochondrial–Lysosomal Axis in Acetaminophen Hepatotoxicity

**DOI:** 10.3389/fphar.2018.00453

**Published:** 2018-05-15

**Authors:** Anna Moles, Sandra Torres, Anna Baulies, Carmen Garcia-Ruiz, Jose C. Fernandez-Checa

**Affiliations:** ^1^Cell Death and Proliferation, Institute of Biomedical Research of Barcelona, Spanish National Research Council, Barcelona, Spain; ^2^Liver Unit, Clinical and Provincial Hospital of Barcelona, Institut d’Investigacions Biomèdiques August Pi i Sunyer and CIBEREHD, Barcelona, Spain; ^3^Research Center for Alcoholic Liver and Pancreatic Diseases, Keck School of Medicine, University of Southern California, Los Angeles, CA, United States

**Keywords:** mitochondria, lysosomes, acetaminophen APAP, mitophagy, lysosomal cholesterol accumulation

## Abstract

Acetaminophen (APAP) toxicity is the most common cause of acute liver failure and a major indication for liver transplantion in the United States and Europe. Although significant progress has been made in understanding the molecular mechanisms underlying APAP hepatotoxicity, there is still an urgent need to find novel and effective therapies against APAP-induced acute liver failure. Hepatic APAP metabolism results in the production of the reactive metabolite *N*-acetyl-*p*-benzoquinone imine (NAPQI), which under physiological conditions is cleared by its conjugation with glutathione (GSH) to prevent its targeting to mitochondria. APAP overdose or GSH limitation leads to mitochondrial NAPQI-protein adducts formation, resulting in oxidative stress, mitochondrial dysfunction, and necrotic cell death. As mitochondria are a major target of APAP hepatotoxicity, mitochondrial quality control and clearance of dysfunctional mitochondria through mitophagy, emerges as an important strategy to limit oxidative stress and the engagement of molecular events leading to cell death. Recent evidence has indicated a lysosomal–mitochondrial cross-talk that regulates APAP hepatotoxicity. Moreover, as lysosomal function is essential for mitophagy, impairment in the fusion of lysosomes with autophagosomes-containing mitochondria may compromise the clearance of dysfunctional mitochondria, resulting in exacerbated APAP hepatotoxicity. This review centers on the role of mitochondria in APAP hepatotoxicity and how the mitochondrial/lysosomal axis can influence APAP-induced liver failure.

## Introduction: Epidemiology and Pharmacology

Acetaminophen [*N*-acetyl-para-aminophenol (APAP)], also known as paracetamol, is one of the most common used drugs worldwide. Since its clinical introduction in 1955, it has become the most widely utilized analgesic/anti-pyretic in many countries around the world. As such, it is estimated that 60 million Americans consume APAP on a weekly basis (Herndon and Dankenbring, 2014). Although APAP is safe when consumed at the recommended doses (<4 g every 24 h), its narrow therapeutic index and its wide over-the-counter availability as a single formulation or in combination with other drugs, makes accidental or deliberate APAP overdose a leading cause of drug-induced liver injury (DILI) in Europe and the United States (Blieden et al., 2014). In the most severe cases of DILI, patients with no previous history of liver disease can develop fulminant liver failure, which usually requires liver transplantation.

Acetaminophen hepatotoxicity is characterized by acute liver failure, centrilobular hepatic necrosis, renal tubular necrosis and hypoglycemic coma (Lancaster et al., 2015), an outcome that can be more frequent with the use of APAP at doses above the therapeutic range. Indeed, ingestion of 15–25 g of APAP is fatal in a quarter of cases due to acute liver failure (Larson et al., 2005). Prospective studies indicated that repeated use of therapeutic APAP dosage may slightly increase the level of serum ALT without manifest acute liver failure (Dart and Bailey, 2007). However, patients taking APAP in the therapeutic range in association with risk factors are likely to develop APAP hepatotoxicity and liver damage. For instance, combination of APAP at therapeutic doses with alcohol intake can result in severe liver injury by the potentiation of APAP metabolism by alcohol along with decreased glutathione (GSH) levels (Zimmerman and Maddrey, 1995). In line with these findings, a potential toxic synergism between APAP and salicylates (e.g., aspirin) can result in liver failure, particularly in children, potentiated by the exhaustion of GSH levels (Dinakaran and Sergi, 2018). Overall APAP intoxication accounts for 40–45% of all cases of acute liver failure (Nourjah et al., 2006) with a mortality rate of approximately 0.4% (450 deaths a year) in the United States (Bunchorntavakul and Reddy, 2013; Yoon et al., 2016).

Therapeutic peak concentrations of APAP (10–20 μg/mL) are detected as quickly as 90 min after its oral ingestion due to its fast absorption in the duodenum (McGill and Jaeschke, 2013). Serum half-life for a healthy individual taking a therapeutic dose ranges from 1.5 to 3 h. However, a prolonged half-life of more than 4 h can occur in individuals that either consume an overdose of APAP or that exhibit a clinical history of hepatic injury or chronic liver disease (Cummings et al., 1967).

As APAP hepatotoxicity is the most common cause of acute liver failure requiring liver transplantation, a better understanding of the molecular events contributing to APAP hepatotoxicity may provide novel opportunities for intervention. While the molecular mechanisms of APAP hepatotoxicity are diverse and include different players, including innate immune response, in this review we will focus on the mitochondrial–lysosomal axis, which has emerged as a novel liason in several pathologies, including lysosomal storage diseases (Torres et al., 2017a), to pinpoint its role in APAP-mediated hepatotoxicity.

## APAP Metabolism: Mitochondrial Targeting

**FIGURE 1:**
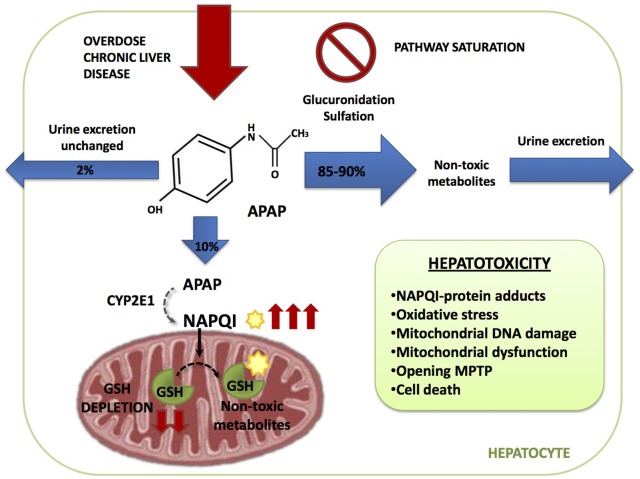
Acetaminophen (APAP) metabolic pathway. APAP is metabolized in the hepatocyte through three different pathways. 2% is excreted through the urine unchanged, 85–90% is converted in glucuronidated and sulfated non-toxic metabolites and 10% is oxidized by cytochrome CYP2E1 generating the highly reactive and toxic metabolite *N*-acetyl-*p*-benzoquinone imine (NAPQI). NAPQI is then conjugated with gluthatione (GSH) resulting in non-toxic metabolites. During APAP overdose or susceptible in patients, as the ones with chronic liver disease, glucuronidation and sulfation pathways become saturated and more APAP is metabolized through CYP2E1 which increases NAPQI generation depleting GSH liver stores. Free unconjugated NAPQI reacts with proteins generating NAPQI-protein adducts in hepatocytes leading to mitochondrial dysfunction and cell death.

Acetaminophen hepatoxicity is caused by the action of a reactive metabolite derived from APAP metabolism in the liver that ultimately targets mitochondrial components resulting in impaired mitochondrial function. APAP metabolism can be divided in three phases (**Figure 1**):

(1) The major bulk of APAP (85–90%) is metabolized by UDP-glucuronosyl transferases (UGT) and sulfotransferases (SULT), with conversion to glucuronidated (56–60%) and sulfated (28–30%) non-toxic metabolites, which are excreted through the urine (Cummings et al., 1967; McGill and Jaeschke, 2013).
(2) A minor fraction of APAP (2–5%) is directly excreted in the urine.
(3) The remainder of APAP (5–10%) is oxidized predominantly by cytochrome P4502E1 (CYP2E1) and to a lesser extent by CYP1A2 and 3A4 to generate the highly reactive and toxic metabolite *N*-acetyl-*p*-benzoquinone imine (NAPQI). At therapeutic APAP doses or when liver GSH levels are high, NAPQI is rapidly conjugated with GSH resulting in non-toxic mercapturic acid and cysteine conjugates, which are secreted in the urine. Small quantities can also be oxidized by myeloperoxidase and cyclooxygenase-1 into non-reactive metabolites (Larson, 2007).

Acetaminophen overdose results in the saturation of the glucuronidation and sulfation pathways. Therefore, excess APAP is predominantly metabolized by CYP2E1, leading to an increased NAPQI output, which may result in the depletion of liver GSH stores. Furthermore, if APAP metabolism occurs in the face of limited GSH levels, such as in the case of alcohol consumption, free unconjugated NAPQI reacts with sulfhydryl groups on cysteine and lysine residues, generating NAPQI-protein adducts (APAP-protein adducts) in hepatocytes, particularly in mitochondria, leading to mitochondrial dysfunction and cell death (Yuan and Kaplowitz, 2013).

The binding of NAPQI to mitochondrial proteins contributes to mitochondrial dysfunction (Lancaster et al., 2015) following mitochondrial GSH (mGSH) depletion, causing the generation of reactive oxygen species (ROS), oxidative stress and mitochondrial DNA damage. Moreover, NAPQI can affect ATP-synthase α-subunit, leading to defective or impaired ATP production (Jaeschke et al., 2012), which contributes to leakage of mitochondrial proteins into the cytosol that promote cell death due to the induction of the mitochondrial permeability transition (MTP) pore (**Figure 1**). MTP opening dissipates the impermeability of the mitochondrial inner membrane (MIM) allowing the transfer of solutes into the matrix, causing secondarily the rupture of the mitochondrial outer membrane (MOM). Therefore, early intervention in replenishing mGSH stores by supplying GSH precursor such as *N*-acetylcysteine (NAC) or targeting upstream mechanisms involved in mitochondrial dysfunction and ROS generation, such as the sustained activation of c-Jun N-terminal kinases (JNK), can result in the protection of APAP-mediated hepatotoxicity.

## APAP Hepatotoxicity: Mitochondrial Oxidative Stress and Amplification by JNK

### Mitochondrial Oxidative Stress

**FIGURE 2:**
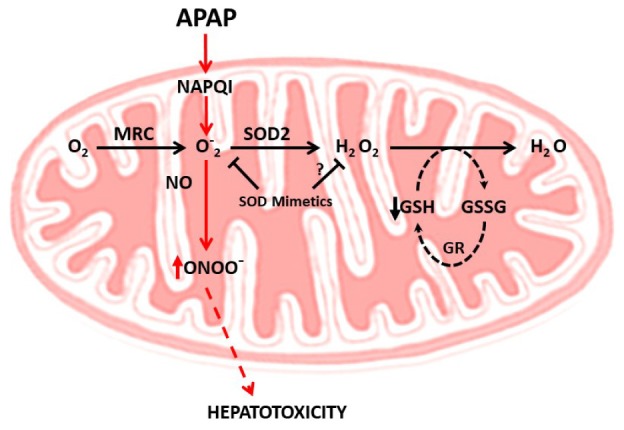
Mitochondrial oxidative and nitrosative stress in APAP hepatotoxicity. APAP metabolism results in NAPQI generation, which induces superoxide anion generation within the mitochondrial respiratory chain (MRC). The combination of superoxide anion with nitric oxide (NO) generates peroxynitrite (ONOO^-^), a free radical which induces nitrosative stress and cell death. The scavenging of superoxide anion by SOD2 decreases ONOO^-^ generation, suggesting the use of SOD mimetics to ameliorate APAP hepatotoxicity. The specificity of SOD mimetics in scavenging superoxide anion vs. hydrogen peroxide may determine protection against APAP hepatotoxicty, particularly in the face of mGSH depletion. In this scenario of decreased mGSH levels superoxide anion scavenging may result secondarily in increased hydrogen peroxide formation, which can lead to enhanced hepatocellular cell death, as reported in the context of non-alcoholic steatohepatitis. Whether this is the case in APAP hepatotoxicity remains to be determined.

As indicated, one of the predominant mechanisms underlying APAP-mediated hepatotoxicity is the induction of mitochondrial oxidative stress. Indeed, during APAP-induced toxicity there is an increased generation of mitochondrial superoxide anion production, stimulated by impaired mitochondrial respiration and formation of adducts between the reactive metabolite NAPQI and sulfhydryls moieties from mitochondrial proteins (Yan et al., 2010). Mitochondrial superoxide anion can react with nitric oxide (NO) generating the reactive radical peroxynitrite (ONOO^-^) and both superoxide anion and ONOO^-^ formation in mitochondria are important events contributing to APAP-induced hepatotoxicity (Ramachandran and Jaeschke, 2017). Free ONOO^-^ then nitrosylates protein tyrosine residues, which in turn can compromise protein function (Radi et al., 2001). The source of mitochondrial NO during APAP-induced hepatotoxicity is still unclear. The mitochondrial NO synthase (mtNOS) has been shown to generate NO and hence ONOO^-^ within mitochondria. Although expression of mtNOS has been described in different contexts, recent evidence has refuted a role for mtNOS in mitochondrial NO generation, at least in rat liver mitochondria (Venkatakrishnan et al., 2009). Moreover, while ONOO^-^ production seems to be independent of inducible nitric oxide synthase (iNOS) (Saito et al., 2010), neuronal NOS (nNOS), which is present in hepatocytes, may play a role as nNOS inhibition or deficiency has been shown to protect against APAP-mediated liver damage (Agarwal et al., 2012; Banerjee et al., 2015). Furthermore, consistent with the role of ONOO^-^ in APAP hepatotoxicity, manganese superoxide dismutase (SOD2) deficient mice exhibit increased liver injury accompanied by enhanced ONOO^-^ and protein carbonylation following APAP challenge (Ramachandran et al., 2011). Thus, these findings suggest that scavenging superoxide anion may be beneficial against APAP-mediated hepatotoxicity by the expected decrease in ONOO^-^ formation (**Figure 2**). In this regard, the mitochondria-targeted SOD mimetic Mito-tempo has been shown to protect against APAP hepatotoxicity, while Tempo, which exhibits SOD activity but without its specific mitochondrial targeting, failed to protect against APAP-induced liver injury (Du et al., 2017), illustrating the relevance of mitochondrial ONOO^-^ generation in APAP hepatotoxicity. Although not reported for Mito-tempo other mitochondria-targeted SOD mimetics such as Mito-CP have been shown to reduce hydrogen peroxide and the subsequent hydrogen peroxide-induced inactivation of complex I and aconitase in endothelial cells (Dhanasekaran et al., 2005). Thus, the specificity of SOD mimetics in scavenging superoxide anion vs. hydrogen peroxide may determine protection against APAP hepatotoxicty, particularly in the face of mGSH depletion. Indeed, in this scenario of decreased mGSH levels superoxide anion scavenging may result secondarily in increased hydrogen peroxide formation, which can lead to enhanced hepatocellular cell death, as reported in the context of non-alcoholic steatohepatitis (von Montfort et al., 2012). In line with this possibility, it has been shown that GSH protects against APAP-induced hepatotoxicity and liver injury in mice through the scavenging of ONOO^-^ (Bajt et al., 2003). However, while mitochondrial nitrosative and oxidative stress contributes to mitochondrial damage, this event alone may not be enough to trigger cell death, requiring further amplification mechanisms.

### Role of JNK

**FIGURE 3:**
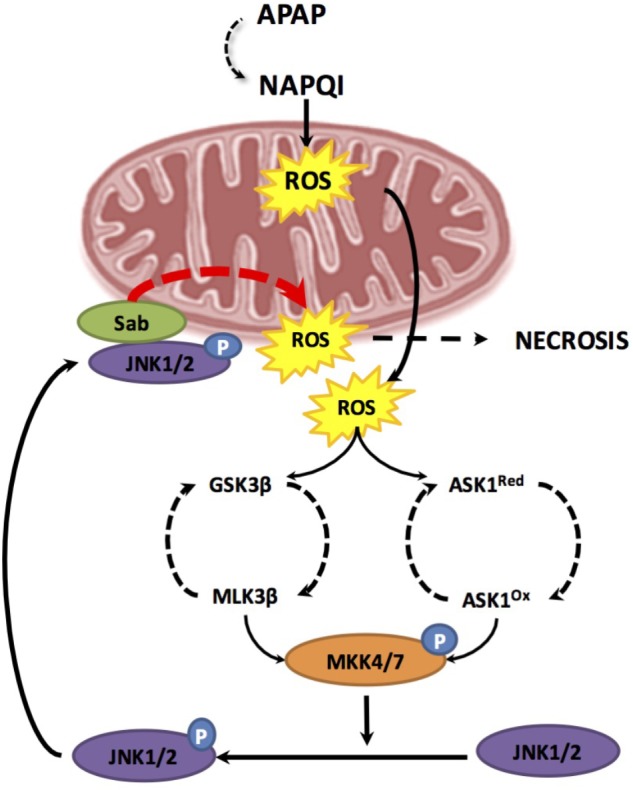
Two hit model during APAP-induced hepatotoxicity. The first hit involves mitochondrial glutathione depletion due to its covalent binding to the reactive metabolite NAPQI resulting from APAP metabolism through CYP2E1. GSH depletion results in enhanced mitochondrial reactive oxygen species (ROS) production. Increase ROS leads to two phases of mitogen-activated protein kinase (MAPK) activation, including MLK3 most likely through GSK-3b and ASK-1. Both pathways result in the activation of MKK4/7 which in turn activates JNK. Once JNK is activated by phosphorylation, it translocates to the mitochondria and binds to Sab. This leads to a sustained production of mitochondrial ROS triggering a positive feedback loop which results in self-amplification of MAPK activation pathways, MPT, mitochondrial failure and necrosis.

As mentioned, NAPQI generation can directly cause oxidative stress and mitochondrial dysfunction following mitochondrial protein adduct formation. However, these initial events may be transient and insufficient to induce hepatocellular death and hence overt liver injury. Therefore, an amplification loop sustaining mitochondrial dysfunction in APAP-mediated hepatotoxicity has been suggested, involving two hits. This model proposes that mGSH depletion due to its covalent binding to NAPQI acts as a first-hit, which is followed by a burst of mitochondrial ROS production that activates a second hit involving mitogen-activated protein kinases (MAPKs) that in turn amplifies the wave of mitochondrial ROS generation (Lemasters, 1998). Consistent with the role as a second hit, MAPK are known to be activated by oxidative stress and have been involved in APAP hepatotoxicity. One of the first MAPK to draw attention in APAP hepatotoxicity is apoptosis signal-regulating kinase1 (ASK1), a mitogen-activated protein kinase kinase kinase (MAP3K), which regulates JNK and p38 pathways. Under unstimulated conditions, ASK1 exists in a high-molecular-mass complex called the signalosome, which comprises an ASK1 homodimer and the reduced form of thioredoxin (Trx). Under oxidative stress and due to its redox responsiveness, ASK1 links ROS generation to JNK activation. Kinetic studies of JNK activation by APAP indicated that ASK1 silencing affects prolonged (>1.5 h) but not initial (≤1.5 h) JNK activation, indicating that ASK1 may only be required for sustained JNK activation after APAP (Du et al., 2015). Thus, during APAP metabolism, NAPQI-mediated oxidative stress leads to MAPK activation, which in turn recruits and activates JNK in mitochondria to amplify APAP hepatotoxicty (Matsumaru et al., 2003) (**Figure 3**).

In line with the link between JNK activation and mitochondrial targeting, there is extensive experimental evidence supporting a direct role for JNK activation in MPT pore opening. Translocation of phosphorylated-JNK (p-JNK) to the mitochondria enhances mitochondrial oxidative stress in mouse and humans (Hanawa et al., 2008; Xie et al., 2014; Du et al., 2015). In this process, translocated p-JNK binds to Sab, a scaffold protein located in MOM, which results in sustained production of mitochondrial ROS, triggering a positive feedback loop of self-amplifying MAPK activation pathways, which culminate in MPT, mitochondrial dysfunction and ultimately necrosis (Han et al., 2013; Du et al., 2015). As recently deciphered, JNK emerges as a key culprit in APAP-induced hepatocellular death and liver damaged caused by a Sab-dependent mechanism that involves inactivation of intramitochondrial Src in the MIM, which in turn inhibits electron transport, increases ROS, and sustains JNK activation (Win et al., 2016). Interestingly, recent findings have shown a link between PKC and JNK activation (Saberi et al., 2014). The pharmacological and genetic antagonism of PKC protected against APAP-mediated liver injury by inhibiting JNK activation, while JNK1 and JNK2 silencing *in vivo* decreased APAP-mediated PKC translocation to mitochondria, unraveling a feed-forward mechanism involved in APAP hepatotoxicity. In addition to the functional link between PKC and JNK, PKC also protects against APAP hepatotoxicity by a JNK-independent mechanism involving autophagy induction, as discussed below.

The role of JNK as an amplification pathway for mitochondrial damage induced by APAP has been extensively investigated using different approaches, from pharmacological inhibition to antisense oligonucleotides-mediated knockdown of *Jnk1* and *Jnk2* or global *Jnk2* deletion in mice (Han et al., 2013; Du et al., 2015; Jaeschke, 2016). Moreover, targeting upstream kinases such as ASK1 or mixed-lineage kinase3 (MLKL3) attenuated JNK activation and liver injury (Han et al., 2013; Du et al., 2015). While a highly specific ASK1 inhibitor effectively prevented JNK activation and protected against APAP hepatotoxicity, the efficacy of the ASK1 inhibitor was lost when administered after JNK was activated and translocated to the mitochondria (Xie et al., 2015), highlighting the hierarchical relationship between ASK1 and JNK in APAP hepatotoxicity. In contrast to upstream kinases that promote JNK activation and liver injury in response to APAP, the MAPK phosphatase, which negatively regulates JNK, protects against APAP hepatotoxicity (Du et al., 2015). Despite these findings involving JNK in APAP hepatotoxicity, a recent report in mice with hepatocyte-specific *Jnk1* and *Jnk2* deletion (Jnk^Δhepa^) questioned the role of JNK in APAP-induced hepatotoxicity (Cubero et al., 2016). The strategy included the crossing of mice with floxed allele of *Jnk1*, generated by homologous recombination in ES cells and backcrossed to the C57BL/6J strain with *Jnk2*-deficient mice to generate *Jnk1*^LoxP/LoxP^/*Jnk2*^-/-^ mice. Crossing these *Jnk1*^LoxP/LoxP^/*Jnk2*^-/-^ mice with Alb-Cre mice produced *Jnk*^Δhepa^ (Cubero et al., 2016). Intriguingly, *Jnk*^Δhepa^ mice developed higher liver injury than wild-type animals after APAP overdose, suggesting a beneficial role for combined Jnk1 and Jnk2 activation in hepatocytes during APAP-induced hepatotoxicity. While these studies provided evidence for a paradigm shift in the role of JNK in APAP hepatotoxicity, there are some reservations that need to be further explored before discarding a role for JNK in APAP-induced liver injury. First, while *Jnk2* was globally deleted in all cell types, *Jnk1* was specifically ablated in hepatocytes but present in non-parenchymal cells, implying an opposing role of Jnk1 in different subsets of liver cells as well as in infiltrated inflammatory cells. Second, crossing *Jnk1*^LoxP/LoxP^/*Jnk2*^-/-^ mice with Alb-Cre mice deletes *Jnk1* in hepatocytes during embryogenesis, raising the possibility of compensatory gene induction that could interfere with the final outcome. Therefore, the use of mice with inducible double knockout for *Jnk1/Jnk2* may be a valuable approach to address the role of JNK in APAP-mediated hepatotoxicity.

## Mitochondrial Damage and Cell Death During APAP-Mediated Hepatotoxicity

As mentioned above, APAP induces MPT opening, an event that was initially associated with apoptotic cell death pathways. However, it is known that MPT can participate in several other forms of cell death (Lemasters, 1998). Consistent with the predominant hepatocellular necrosis, APAP-induced MPT may be a crucial cellular event contributing to hepatocyte death and liver injury during APAP hepatotoxicity.

The onset of MPT is characterized by the permeabilization of the MOM secondary to a sudden increase in permeability of the MIM, which allows the exit of molecules less than 1500 Da (Karch et al., 2013). The molecular composition of MPT operating at both MOM and MIM are not well-understood at present, despite evidence indicating a role for voltage-dependent anion channel (VDAD) and adenine nucleotide translocase (ANT) in the regulation of MPT. In addition, the components of MIM that participate or determine MPT remains controversial to date (Karch and Molkentin, 2014). In contrast to VDAC and ANT, cyclophillin D has been shown to be a key constituent and determinant of MPT (Nakagawa et al., 2005; McCommis and Baines, 2012; Sileikyte et al., 2014; Gutierrez-Aguilar and Baines, 2015).

Oxidative stress induces MPT by targeting specific cysteine residues of cyclophillin D, which results in mitochondrial depolarization, oxidative phosphorylation uncoupling, release of intra-mitochondrial ions and metabolic intermediates, mitochondrial swelling and decrease in ATP synthesis. Oxidants such as Ca^2+^ and peroxides promote MPT during APAP-induced hepatotoxicity (Chen et al., 2008). MPT inhibitors such as cyclosporine A, trifluoperazine, or dithiothreitol protect against APAP hepatotoxicity by preventing loss of mitochondrial membrane potential and a further burst in oxidative stress (Reid et al., 2005). However, MPT inhibitors do not stop NAPQI generation or GSH depletion (Kon et al., 2004, 2007).

The permeabilization of the MOM due to MPT releases apoptogenic proteins such as cytochrome c, second mitochondria-derived activator of caspase (Smac), apoptosis inducing factor (AIF), and endonuclease G from the intermembrane space. Although the release of these proteins into the cytosol is expected to trigger apoptosis, this form of cell death is negligible in APAP-induced hepatotoxicity, as shown by the absence of caspases activation and the inability of caspase inhibitors to protect against APAP-induced liver injury (Jaeschke et al., 2006). Consistent with this event, the release of both AIF and endonuclease G contribute to caspase-independent cell death by translocating into the nuclei triggering DNA fragmentation (Bajt et al., 2006). Morphological characteristics of necrosis such as organelle swelling, release of cell components and karyolysis are evident in hepatocytes after APAP injury (Jaeschke et al., 2004). Therefore, hepatotoxicity induced by APAP is largely necrotic and caspase independent (Jaeschke et al., 2006, 2011). Opening of the MPT pore in the MIM results in necrosis due to energy dissipation and ion pump/channel failure. In addition, as caspases are oxidant stress sensitive exhibiting dual regulation by ROS, it is conceivable that overt oxidative stress and ROS generation may block caspase activation despite release of apoptosis factors from mitochondria. Although necroptosis, a regulated form of necrosis, has been speculated to possibly mediate APAP-induced liver injury, its contribution to APAP hepatotoxicity is still unclear (Dara et al., 2015, 2016). Moreover, although it is well-described that APAP-induced hepatotoxicity is mostly necrotic, switch toward apoptotic cell death has been shown *in vitro* after treatment with APAP and fructose, which acts as a glycolytic substrate to generate ATP and glycine as a membrane stabilizer (Kon et al., 2004). Therefore, the relative amount of ATP seems to be key to determine the preferential mode of cell death of hepatocytes after APAP challenge.

As MPT and JNK are key players in APAP-mediated acute liver failure, it is unclear whether mitochondrial dysfunction and JNK are engaged in low dose APAP exposure without liver injury. In comparing high (300 mg/Kg) vs. low (150 mg/Kg) APAP, Hu et al. (2016b) showed the reversible onset of MPT and mitochondrial dysfunction in the absence of liver necrosis in response to low but not high APAP dosing. Mitochondrial protein NAPQI adducts correlated with early and transient JNK activation, but irreversible mitochondrial depolarization and necrosis at high dose were associated with sustained JNK activation and translocation to mitochondria. MPT inhibition by NIM811 prevented cell death and/or mitochondrial depolarization after both high and low dose APAP, while the JNK inhibitor SP600125 decreased mitochondrial depolarization after low dose. Thus the reversible vs. the irreversible MPT-dependent mitochondrial dysfunction and sustained mitochondrial JNK activation determine APAP-mediated liver necrosis. These findings indicate that as opposed to toxic APAP exposure non-toxic APAP challenge can cause transient mitochondrial dysfunction, which is insufficient to promote liver damage.

## Lysosomes and APAP Hepatotoxicity: Lysosomal Instability and Iron Release

While the role of mitochondria during APAP-induced hepatotoxicity has been extensively studied and reviewed (Masubuchi et al., 2005; Latchoumycandane et al., 2007; Pessayre et al., 2012; Han et al., 2013; Ni et al., 2015; Ramachandran and Jaeschke, 2017), the role of lysosomes in APAP hepatotoxicity has been less investigated and currently is poorly understood. Lysosomal instability and release of lysosomal content into the cytosol have been proposed as a mechanism by which lysosomes can contribute to APAP-induced hepatotoxicity. For instance it has been shown that APAP can increase the activity and the release of lysosomal proteases, such as cathepsin B (Woolbright et al., 2012) and D (Khandkar et al., 1996). As cathepsin B has been previously reported to play a significant role in promoting cell death during liver injury (Guicciardi et al., 2000), it may be anticipated that APAP-mediated release of lysosomal cathepsin B may contribute to APAP-induced liver injury. Quite intriguingly, however, cathepsin B inhibition during APAP-induced injury did not show a significant effect, suggesting that the mobilization of cathepsin B from lysosomes into cytosol does not play *per se* a role in APAP hepatotoxicity (Woolbright et al., 2012). On the other hand, lysosomal cathepsin D release may have the potential to regulate APAP hepatotoxicity by modulating the proteolytic degradation of cytochrome P450. In this regard, it has been shown that anti-cathepsin D neutralizing antibody decreased APAP-induced degradation of CYP3A4 (Zhang et al., 2004). However, the relevance of these findings and hence of the role of lysosomal cathepsin D in *in vivo* APAP-mediated acute liver failure is uncertain given the minor role of CYP3A4 in APAP metabolism (see above).

While these results suggest that lysosomal cathepsins do not appear to play a role in APAP-induced hepatotoxicity, lysosomes are an important source of iron, a catalyst known to stimulate ROS generation. Thus, lysosomes could modulate APAP hepatotoxicity via iron regulation and subsequent oxidative stress. Iron exists in two different pools in the liver. The non-chelatable iron pool is sequestered in ferritin and in specific proteins [e.g., heme and iron–sulfur complexes (ISCs)] as an structural component, and therefore this iron pool cannot be removed by conventional iron chelators, like desferal. The second pool is chelatable iron, which includes free iron and the iron loosely bound to a wide range of anionic intracellular molecules. Previous studies indicated that lysosomal iron is released and translocated into the mitochondria contributing to MPT in APAP-induced injury (Kon et al., 2010). While the mechanisms underlying APAP-mediated lysosomal instability or permeabilization are not fully understood, the iron chelator desferal prevented mitochondrial depolarization and hepatocellular death after APAP exposure (Kon et al., 2010). In analyzing the cross-talk between lysosomes and mitochondria, it has been shown that the mitochondrial Fe^2+^ uniporter MCFU plays a key role in APAP-induced oxidative stress, mitochondrial dysfunction, and hepatocellular death (Hu et al., 2016a). Accordingly, inhibiting MCFU by Ru360 or minocycline prevented APAP-induced MPT and hepatocellular death. Iron chelation by starch-desferal prevented MPT and ROS generation. Importantly, minocycline was effective after APAP treatment, emerging as a potential therapeutic agent against APAP hepatotoxicity. Therefore, these studies revealed that the release of Fe^2+^ from lysosomes followed by uptake into mitochondria via MCFU is a key mechanism in APAP-mediated hepatotoxicity. Another mechanism by which the endo/lysosomal compartment can influence APAP-induced hepatotoxicity is through the clearance of damaged mitochondria in a process also called mitophagy, consisting in the degradation of dysfunctional mitochondria by lysosomal proteases upon fusion of the autophagosomes-containing mitochondria with lysosomes, which is discussed in the next section.

## Lysosomes and APAP Hepatotoxicity: Role of Mitophagy and Cholesterol

Autophagy is a complex catabolic process responsable for the degradation and turnover of organelles and cellular debris, which are digested within lysosomes by lysosomal proteases. Autophagy is highly regulated by multiple specialized proteins that govern different stages of this multistep process, including the fusion of autophagosomes with lysosomes. Considered an alternative energy source by the recycling of cellular materials autophagy often acts as a survival process not only by providing energy but in addition by removing dysfunctional organelles, including mitochondria by a specialized process called mitophagy. The mechanisms and regulation of autophagy and mitophagy have attracted considerable attention due to their impact in health and disease and have been recently reviewed (Czaja et al., 2013; Ni et al., 2013). Consistent with this function, it has been shown that APAP induces autophagy both in primary mouse hepatocytes and intact liver and therefore the pharmacological promotion of autophagy by rapamycin protects against APAP hepatotoxicity (Igusa et al., 2012; Ni et al., 2012a, 2013). In this regard, using broad-spectrum PKC inhibitors, it has been shown that PKC blockade upregulated p-AMPK levels, resulting in increased levels of LC3-II formation and p62 degradation indicative of increased autophagy, which resulted in the protection against APAP hepatotoxicity (Saberi et al., 2014), thus complementing the JNK-dependent contribution of PKC in APAP hepatotoxicity described above. Quite intriguingly, liver specific deletion of *Atg5*, an essential gene that regulates autophagy, has been shown to protect against APAP-induced liver injury due to compensatory mechanisms (Ni et al., 2012b). The ablation of *Atg5* suppresses autophagy and p62 degradation, which results in the persistent activation of nuclear factor (erythroid-derived 2)-like 2 (NFE2L2) by releasing the inhibition of KEAP1 on NFE2L2. Increased NFE2L2 activation, in turn, upregulates the synthesis of GSH, which detoxifies NAPQI, and hence protects against APAP hepatotoxicity.

Mitochondria are dynamic organelles, which undergo changes in morphology. As mitochondria function is determined in part by mitochondrial dynamics, the balance between fusion and fission and the expression of proteins that regulate each process can participate in the preservation of cellular integrity and determine APAP hepatotoxicity (Karbowski et al., 2002; Wang et al., 2015; Wai and Langer, 2016; Ramachandran and Jaeschke, 2017). For instance, during APAP hepatotoxicity, expression of dynamin related protein 1 (Drp1), a GTPase that controls mitochondrial fission, increases and translocates into the mitochondria pointing toward an important role for mitochondrial fission in APAP-induced injury (Ramachandran et al., 2013). Moreover, the interaction between Bax and Drp-1 has been involved in APAP hepatotoxicity by facilitating mitochondrial fission, which in turn can initiate downstream events, including opening of the MTP. Interestingly, Drp-1 translocation to mitochondria, which has been suggested to be downstream of JNK activation, is suppressed by RIPK1 or Sab knockdown (Dara et al., 2015). However, Sab silencing did not prevent APAP-induced RIPK1 translocation to mitochondria, suggesting a hierarchichal ordering of events involved in mitochondrial targeting, in which JNK is downstream of RIPK1 and upstream of Drp-1. Unlike RIPK1, the role of RIPK3 in APAP hepatotoxicity is controversial (Ramachandran et al., 2013; Dara et al., 2015) and requires further investigation.

**FIGURE 4:**
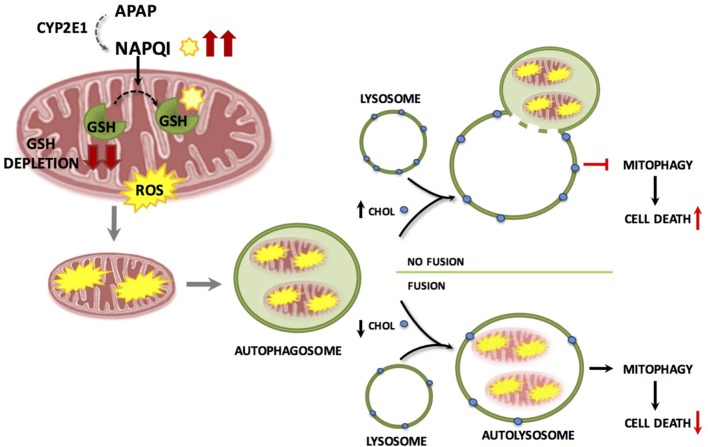
Accumulation of lysosomal cholesterol impairs mitophagy resulting in increased hepatocyte cell death and liver injury during APAP-induced liver injury. APAP metabolism and NAPQI generation and GSH depletion are independent of lysosomal cholesterol content and cause mitochondrial damage and dysfunction. Damaged mitochondria are engulfed by autophagosomes which fuse with lysosomes clearing dysfunctional mitochondria through mitophagy resulting in a decrease in hepatocyte cell death. Accumulation of cholesterol in the lysosomal membrane results in impaired autophagosome/lysosome fusion resulting in defective mitophagy and increased hepatocyte cell death.

The specific removal of dysfunctional mitochondria through mitophagy is determined by several mechanisms, including the mitochondrial translocation of Parkin, an E3 ubiquitin ligase (Youle and Narendra, 2011; Williams et al., 2015). However, the role of Parkin in APAP hepatotoxicity is uncertain as acute Parkin knockdown exacerbates APAP-mediated liver injury while chronic deletion using knockout mice results in resistance to APAP hepatotoxicity, most likely due to compensatory mechanisms that remain to be further characterized (Williams et al., 2015). During mitophagy damaged mitochondria are cleared through fusion of autophagosomes-containing mitochondria with lysosomes. However, little is known about how this fusion process is regulated and controlled during APAP-induced liver injury. Recent findings have shown that lysosomal cholesterol accumulation exacerbates APAP-induced hepatotoxicity and liver injury by impairing mitophagy without increasing APAP metabolism or NAPQI generation (Baulies et al., 2015). The increase in lysosomal cholesterol likely decreases lysosomal membrane dynamics, which in turn reduces the transition from liquid-ordered to liquid-disordered phases. This may contribute to the defective fusion of lysosomes with autophagasomes containing mitochondria (**Figure 4**). Of potential relevance, it has been shown that tricyclic antidepressants like amytriptiline, sensitized against APAP hepatoxicity by inhibiting acidic sphingomyelinase (ASMase), which resulted in lysosomal cholesterol accumulation. In agreement, ASMase knockout mice exhibited enhanced hepatotoxicity and liver damage after APAP overdose, while hepatocytes challenged with U18666A, which induces the accumulation of cholesterol in lysosomes, sensitized hepatocytes to APAP-mediated cell death. In addition to the findings with ASMase knockout mice, which models the lysosomal storage disorder Niemann–Pick type A disease, inhibition of glucocerebrosidase in hepatocytes by conduritol β-epoxide, a cellular model of Gaucher disease that exhibits lysosomal cholesterol accumulation sensitizes to APAP-induced cell death (Baulies et al., 2015). Conversely, reduction of lysosomal cholesterol using 25-hydroxycholesterol, an oxysterol, which acts as a ligand for liver X receptors and suppresses sterol synthesis, decreases cholesterol accumulation and protected ASMase knockout mice against APAP-induced injury. Thus, these findings suggest that the use of antidepressants such as amitriptyline or its analog desipramine should be considered as a risk factor for the consumption of APAP, and also indicate that patients suffering lysosomal storage diseases, such as Niemann–Pick type A/B disease or Gaucher disease may be at risk from developing acute liver failure upon APAP usage. Moreover, free cholesterol accumulation in liver sinusoidal endothelial cells (LSECs) has been shown to exacerbate APAP hepatotoxicity via TLR9 signaling (Teratani et al., 2017). The accumulation of free cholesterol in LSEC endolysosomes disrupted Rab7 membrane trafficking and impaired the transport of TLR9 from late endosomes to lysosomes, enhancing activation of the TLR9/inflammasome pathway mediating APAP-induced liver injury. Hence, free cholesterol loading not only stands as an important player in obesity and chronic fatty liver disease but may underlie the risk of non-alcoholic fatty liver disease as a risk factor for DILI (Massart et al., 2017). In particular, lysosomal cholesterol accumulation may predispose to APAP hepatotoxicity by a dual mechanism involving the impairment of mitophagy in hepatocytes and the disruption of TLR9 trafficking in LSEC. Besides lysosomal cholesterol accumulation, mitochondrial cholesterol loading has been shown to sensitize to TNF/Fas-induced steatohepatitis due to mGSH depletion (Mari et al., 2006), and hence it is conceivable that the intracellular cholesterol trafficking to different compartments (i.e., lysosomes and mitochondria) determines APAP hepatotoxicity by different mechanisms including oxidative stress/mitochondrial dysfunction and impaired mitophagy.

## Concluding Remarks and Future Directions

Acetaminophen hepatotoxicity is responsible for almost half of the cases of acute liver failure in the United States and remains the leading cause for liver transplantation. Therapeutic interventions offered to these patients are still very limited. Better understanding of the cellular and molecular pathways driving APAP-induced liver injury is key to find novel biomarkers, therapeutic targets, and unrecognized drugs which may sensitize patients to APAP-induced hepatotoxicity. Mitochondria play a central role in APAP-induced cell death and injury and therefore greater knowledge about the relationship between mitochondria with other organelles such as lysosomes can result in improved future therapeutic options for APAP-induced liver injury. In addition to the current clinical interventions available to manage APAP hepatotoxicity, including gastrointestinal track decontamination, NAC administration and liver transplantation, the improvement of mitophagy may stand as a promising novel approach for the management of APAP hepatotoxicity. Furthermore, intracellular cholesterol disruption can be regarded as an emerging factor that can increase the risk of therapeutic misadventure from APAP use. In line with this possibility, conditions that result in increased cholesterol accumulation in lysosomes such as the use of tricyclic antidepressants can potentiate APAP hepatotoxicity. Although NAC is often used in the clinic to restore hepatocellular GSH levels to protect against APAP hepatotoxicity, in the context of mitochondrial cholesterol accumulation, which impairs the transport of cytosol GSH into mitochondria, the use of NAC may be inefficient to specifically replenish mGSH stores, requiring the use of permeable GSH prodrugs such as GSH ethyl ester (GSHEE). Indeed, this scenario has recently been explored in NPC disease and may be worth to be investigated in APAP hepatotoxicity (Torres et al., 2017b). Whether combination of autophagy inducers and GSHEE can arise as an effective approach for APAP-induced liver failure remains to be further investigated.

## Author Contributions

AM, ST, and AB revised the literature and conceived the figures. CG-R and JF-C discussed reported findings and conceived the focus of the review. AM, CG-R, and JF-C wrote the manuscript.

## Conflict of Interest Statement

The authors declare that the research was conducted in the absence of any commercial or financial relationships that could be construed as a potential conflict of interest.
